# Effects of postoperative electrical stimulation on quadriceps muscular atrophy in patients with incomplete cervical spinal cord injury. A retrospective study

**DOI:** 10.3389/fneur.2026.1841524

**Published:** 2026-06-19

**Authors:** Lusha Wu, Liansong Lu, Gangqiang Jiang, Yingzhi Xie, Yongjie Gu, Zhenshan Yuan

**Affiliations:** 1 Women and Children’s Hospital of Ningbo University, Ningbo, China; 2 Ningbo No. 6 Hospital, Ningbo Clinical Research Center for Orthopedics, Sports Medicine and Rehabilitation, Ningbo, Zhejiang, China

**Keywords:** electrical simulation, incomplete cervical spinal cord injury, postoperative rehabilitation, quadriceps muscle atrophy, retrospective study

## Abstract

**Background:**

Lower limb disuse atrophy often occurs in patients with incomplete cervical spinal cord injury (SCI) during peri-operative periods. Electrical stimulation (ES) is a novel method that can benefit muscular atrophy. This study aimed to determine whether ES is the most effective method for postoperative management of muscular atrophy in patients with incomplete cervical SCI.

**Methods:**

A total of 80 incomplete cervical SCI patients with single-sided lower limb myasthenia were enrolled in this study from April 2020 to April 2025. Of these, 40 patients were treated with electrical stimulation (ES group), and the remaining 40 patients were treated with traditional physiotherapy (P group). Data were collected from their medical records. Baseline characteristics, quadriceps muscle thickness, and muscle strength grading were measured at the beginning of the study and again at the 2-week follow-up.

**Results:**

Preoperatively, there were no significant differences in age, gender, height, weight, quadriceps muscle thickness, or muscle strength grading between the two groups (*p* > 0.05). This study found that quadriceps muscle thickness in the two groups significantly decreased at 1- and 2-week follow-up visits. However, quadriceps muscle thickness in the ES group was significantly greater than that in the P group (*p* < 0.05).

**Conclusion:**

ES could be a safe and effective postoperative treatment for muscle disuse atrophy in patients with incomplete cervical SCI; however, further research is needed to prove its efficacy.

## Introduction

Spinal cord injury (SCI) is an increasingly prevalent condition, particularly due to its rapid development. The reported incidence of incomplete SCI in East Asia is 5.4 cases per million population annually (1). The cervical spinal cord is a critical neural pathway that connects the brain to the trunk and limbs. Incomplete cervical spinal cord injuries can lead to varying degrees of motor and sensory dysfunction below the level of injury, with lower limb impairment commonly resulting in muscle weakness, limited mobility, and paralysis (2). During the perioperative period, patients are particularly susceptible to lower limb atrophy due to neurological deficits, pain, immobilization, and significantly reduced physical activity levels. The quadriceps muscle, which is the primary extensor of the knee joint, is particularly affected by this atrophy (3). This loss of muscle mass can impair limb function, delay recovery, and increase the risk of complications such as deep-vein thrombosis, osteoporosis, joint contractures, and pressure ulcers (PUs), significantly worsening the quality of life and long-term prognosis (4). In individuals with SCI, the progressive loss of muscle mass not only contributes to reduced survival rates but also results in a decline in muscle density. This decrease in muscle density compromises the natural cushioning effect over bony prominences and impairs local tissue perfusion, thereby creating a favorable environment for the development of PUs (5).

Traditional rehabilitation for muscle atrophy following an injury primarily involves physiotherapy, including passive range-of-motion exercises, manual therapy, and progressive active training, as tolerated by the patient (6). However, during the early postinjury phase or in cases of severe neurological impairment, limited voluntary movement often reduces the effectiveness of conventional therapies (7).

Neuromuscular electrical stimulation (ES) has emerged as a promising adjunctive intervention in neurorehabilitation (8). ES utilizes low-frequency electrical currents delivered through surface electrodes to stimulate target muscles or peripheral nerves, inducing contractions that mimic physiological movement. Evidence suggests that ES may delay or reverse muscle atrophy caused by denervation and immobilization. It can also preserve muscle mass, improve local blood circulation, and potentially enhance recovery in the central nervous system through sensory-motor feedback mechanisms (9).

Despite these potential benefits, the majority of existing studies have focused on complete SCI or chronic rehabilitation settings (10). Clinical evidence remains limited regarding the efficacy and safety of ES during the postoperative subacute phase—when muscle atrophy progresses rapidly—in patients with incomplete cervical SCI (11). Therefore, this retrospective study aimed to compare the effects of ES and conventional physiotherapy on quadriceps muscle atrophy in patients with incomplete cervical SCI. The study hypotheses are as follows: (1) Compared to conventional physiotherapy, postoperative neuromuscular ES would result in reduced quadriceps muscle atrophy in patients with subacute incomplete cervical SCI, and (2) the ES group would demonstrate greater improvement in quadriceps muscle strength than the physiotherapy group (P group) over the same 2-week postoperative period.

## Materials and methods

### Study design

Between April 2020 and April 2025, 80 patients with quadriceps muscle atrophy secondary to incomplete cervical SCI were screened and enrolled in this retrospective study, after receiving approval from the institutional ethics committee. The inclusion criteria were as follows: (1) a diagnosis of unilateral quadriceps muscle atrophy linked to incomplete cervical SCI, (2) age >25 years, and (3) no history of neurological or musculoskeletal diseases. The exclusion criteria were as follows: (1) pacemaker implantation due to cardiac disease, (2) history of trauma or surgery, (3) severe renal dysfunction affecting physical function, (4) refusal to participate in this study, and (5) lower limb deformity or a history of neuromuscular injuries.

### Electrical stimulation therapy

**Figure 1:**
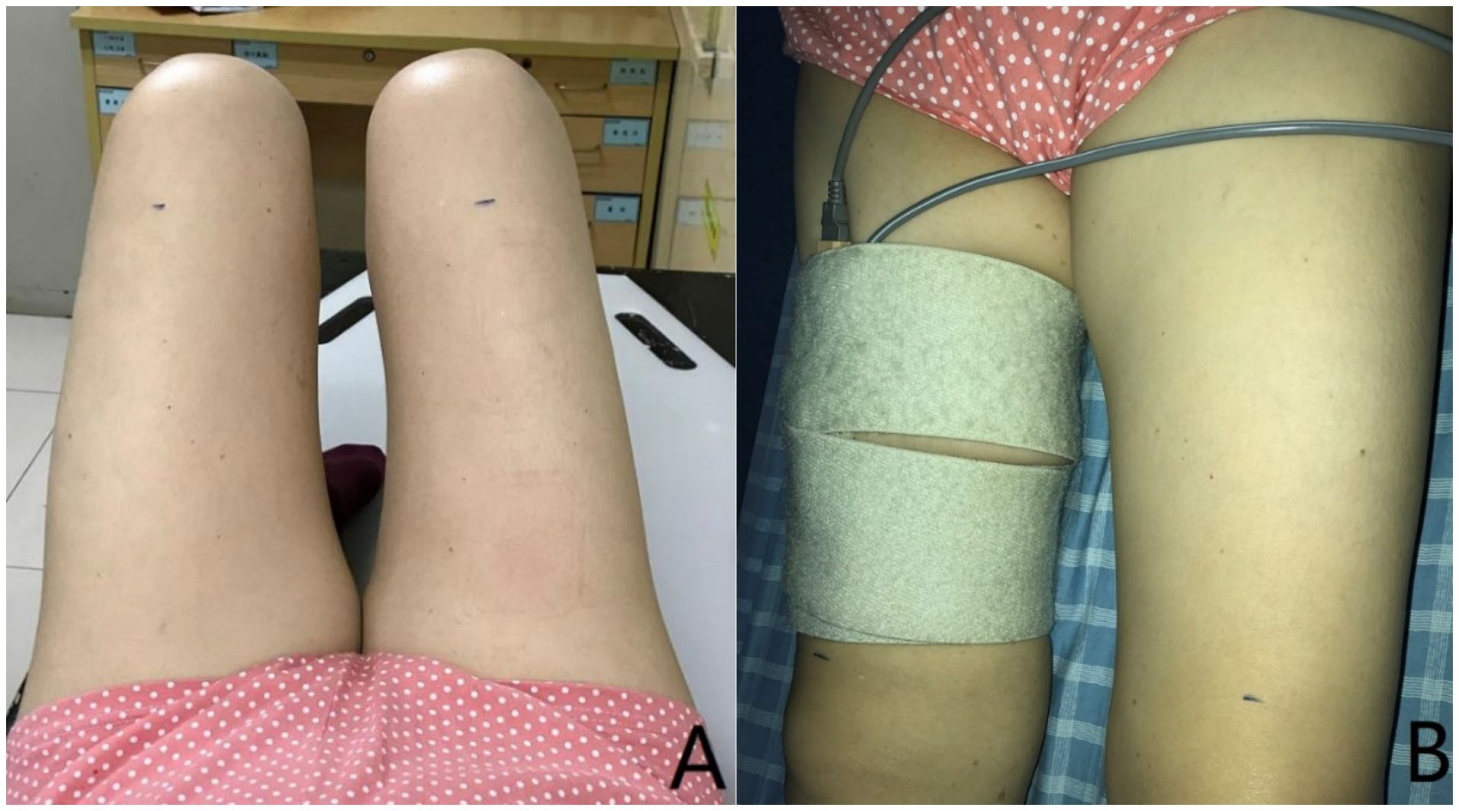
**(A)** Quadriceps muscle atrophy in the right leg, with the measurement point for the cross-sectional perimeter located at the upper middle third of the thigh. **(B)** Electrode placement over the atrophied quadriceps muscle.

The experimental group received standardized rehabilitation supplemented with adjunctive ES using the KD-2A stimulator (Yaoyang Kangda, China). Standardized skin preparation of the lower limb was conducted prior to treatment (12). ES was applied to the quadriceps muscles twice daily during the first 2 weeks postoperatively, following the initial measurement of the muscle cross-sectional area. Before each session, the lower limbs were shaved, and rectangular electrodes (90 × 50 mm) were placed over the atrophied quadriceps muscle and secured with bandaging (Figure 1). The stimulation parameters included biphasic symmetric impulses at a frequency of 50 Hz, a voltage of 220 V, a pulse duration of 0.3 ms, and a duty cycle of 12 s on and 6 s off. The intensity was adjusted to elicit visible muscle contractions while remaining tolerable. Each session lasted 45 min.

### Physiotherapy

All patients received 30 min of traditional rehabilitation therapy, including massage, acupuncture, and conventional physiotherapy, for five sessions each week to help alleviate numbness and pain. Moreover, continuous passive motion therapy was also administered while the patients were in a supine position, with limb movement set at 20–50 cycles/min. The duration and continuation of each session were determined by the treating physician based on the individual’s condition and the severity of their symptoms.

### Measurement of outcomes

Patients were evaluated in the supine position, with their hips and knees fully extended, following a relaxed resting state. Quadriceps muscle thickness was measured using ultrasound on the first postoperative day, serving as the baseline measurement, and was reassessed on postoperative days 7 and 14. Muscle strength and MacNab functional outcomes were recorded concurrently. According to the MacNab criteria (13, 14), outcomes were classified as excellent, good, fair, or poor. Preoperative neurological status was assessed using the American Spinal Injury Association (ASIA) impairment scale (15).

### Statistical analysis

Statistical analysis was conducted using SPSS for Windows version 20 (IBM, Chicago, IL, United States). Continuous data were expressed as mean ± standard deviation. Demographic data and baseline characteristics were analyzed using the chi-square tests or independent *t*-tests, as appropriate. Within-group changes were assessed using paired *t*-tests, while between-group differences were analyzed using independent *t*-tests. A *p*-value of <0.05 was considered statistically significant.

## Results

**Figure 2:**
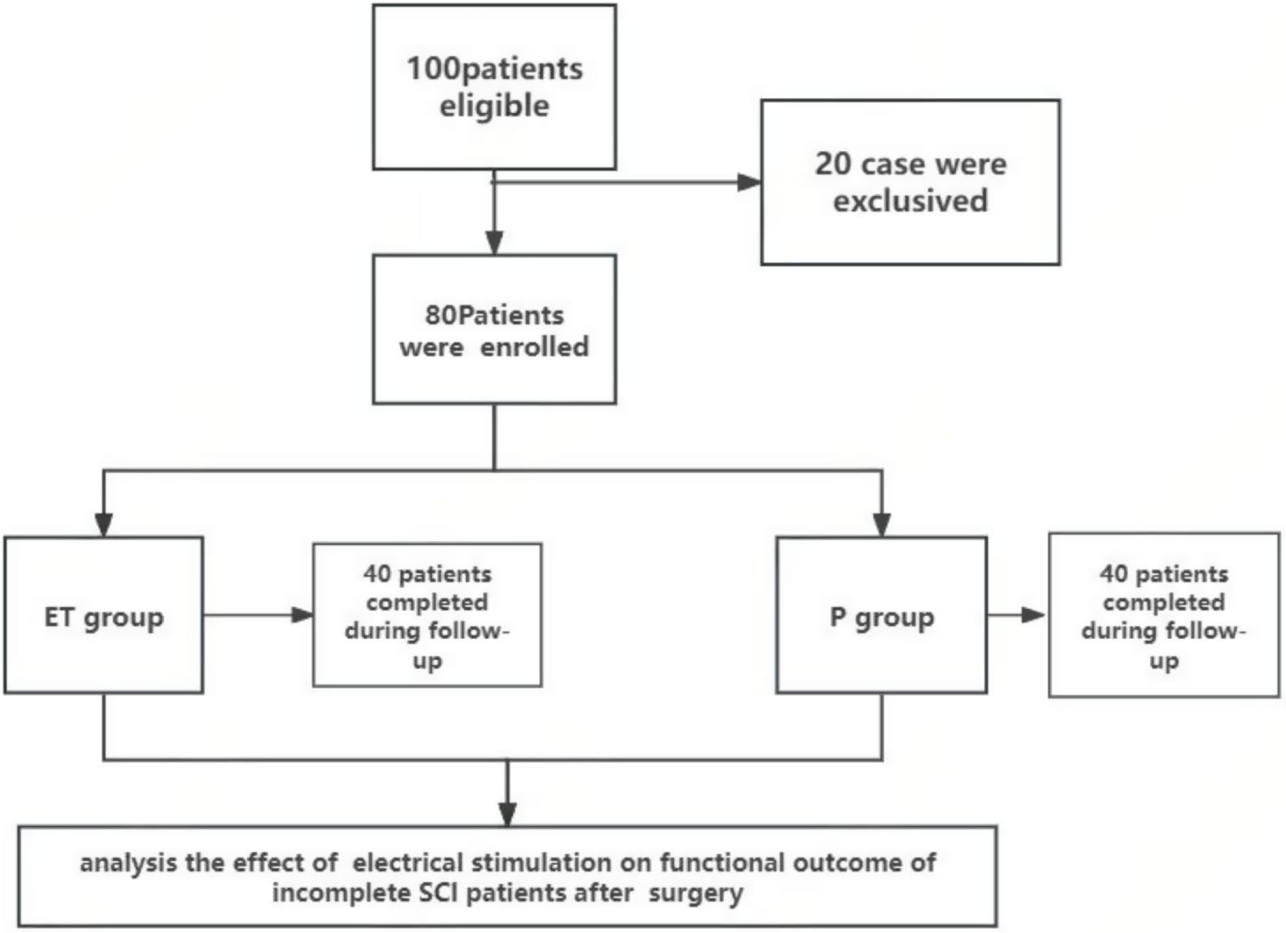
Flowchart of participants involved in the study.

A total of 100 patients with unilateral quadriceps muscle atrophy secondary to incomplete cervical SCI who underwent cervical surgery met the inclusion criteria. Fifteen patients were excluded due to their refusal to participate, and five were excluded due to comorbid conditions that affected the evaluation of outcomes. The final cohort consisted of 80 patients, with 40 patients in the ES group and 40 patients in the P group. All patients completed their follow-up and were included in the analysis (Figure 2).

### Demographic data

No significant differences in baseline demographic or clinical characteristics were observed between the groups (*p* > 0.05). Variables including age, gender, height, weight, body mass index, and ASIA scale scores were comparable between groups (Table 1). The time to initiate walking was significantly shorter in the ES group compared to the P group (7.6 ± 1.2 days vs. 8.9 ± 1.8 days; *p* < 0.05).

**Table 1:** Baseline characteristics of the participants.

Characteristics	ES group (*n* = 40)	P group (*n* = 40)	*p*
Age (years)	49.7 ± 13.1	53.2 ± 8.1	0.15
Height (m)	1.72 ± 0.4	1.69 ± 0.5	0.12
Weight (kg)	76.4 ± 9.3	74.9 ± 10.1	0.51
Body mass index	25.9 ± 3.5	26.1 ± 4.2	0.87
Gender			
Men	30	32	0.39
Women	10	8	
Days to start walking	7.6 ± 1.2	8.9 ± 1.8	<0.05
ASIA scale			
C	24	22	0.21
D	16	18	

ASIA, American spinal injury association impairment scale.

### Clinical outcomes

The outcomes demonstrated significantly greater improvement in muscle thickness in the ES group compared to the P group at both 1- and 2-week follow-up visits (*p* < 0.05). No significant between-group differences were observed in the MacNab classification or serum albumin levels (*p* > 0.05). Preoperative lower limb muscle strength was comparable between the groups, with no clinically significant differences observed (weaker side: 3.0 ± 0.8 vs. 2.9 ± 0.8, *p* < 0.05; stronger side: 2.5 ± 0.4 vs. 2.4 ± 0.4, *p* < 0.05). At both 1- and 2-week follow-up visits, the ES group showed significantly greater improvements in muscle thickness and muscle strength than the P group (*p* < 0.05; Table 2).

**Table 2:** Comparison of clinical outcomes between the two groups.

Characteristics	ES group (*n* = 40)	P group (*n* = 40)	*p*
MacNab criteria			
Excellent	20	19	0.19
Good	12	11	
Fair	7	8	
Poor	1	2	
Serum albumin levels			
Pre	39.2 ± 2.1	39.6 ± 2.4	0.39
1 week	37.1 ± 1.6	37.3 ± 2.1	0.66
2 weeks	37.5 ± 1.5	38.3 ± 2.2	0.06
Muscle thickness (mm)			
Weaker side			
Pre	32.4 ± 0.6	32.6 ± 0.7	0.17
1 week	31.1 ± 0.7	30.3 ± 0.3	<0.05
2 weeks	31.5 ± 0.7	30.9 ± 1.0	<0.05
Stronger side			
Pre	32.5 ± 0.6	32.6 ± 0.8	0.63
1 week	32.0 ± 0.5	31.4 ± 0.6	<0.05
2 weeks	32.8 ± 0.5	32.1 ± 0.6	<0.05
Muscle strength grading			
Weaker side			
Pre	2.5 ± 0.5	2.5 ± 0.5	0.83
1 week	3.5 ± 0.5	2.9 ± 0.9	<0.05
2 weeks	4.5 ± 0.6	4.2 ± 0.7	<0.05
Stronger side			
Pre	3.0 ± 0.8	2.9 ± 0.8	0.79
1 week	3.6 ± 0.5	3.4 ± 0.7	0.01
2 weeks	3.9 ± 0.9	3.5 ± 0.5	<0.05

To better characterize the treatment effects, changes in muscle thickness and muscle strength were analyzed at each follow-up assessment (Table 3). The ES group demonstrated significantly greater improvement in the atrophied limb compared to the P group at all follow-up assessments. In contrast, no significant recovery was observed in the non-atrophied limb in either group throughout the follow-up period (*p* > 0.05).

**Table 3:** Comparison of changes in muscle thickness and muscle strength between the two groups.

Characteristics	ES group (*n* = 40)	P group (*n* = 40)	*p*
Muscle thickness			
Weaker side			
1 week	−0.49 ± 0.81	−1.24 ± 1.2	<0.05
2 weeks	−1.08 ± 0.68	−1.24 ± 0.82	<0.05
Stronger side			
1 week	−0.37 ± 1.01	−1.32 ± 0.73	<0.05
2 weeks	−0.02 ± 0.84	−1.57 ± 0.56	<0.05
Muscle strength grading			
Weaker side			
1 week	0.7 ± 0.39	0.5 ± 1.1	0.03
2 weeks	0.4 ± 0.2	0.2 ± 0.1	0.04
Stronger side			
1 week	0.93 ± 0.2	0.38 ± 0.2	<0.05
2 weeks	0.71 ± 0.4	0.47 ± 0.3	<0.05

### Changes in quadriceps muscle thickness

At the 1-week follow-up, changes in quadriceps muscle thickness were −0.37 ± 1.01 on the stronger side and −0.49 ± 0.81 mm on the weaker side in the ES group, compared to −1.32 ± 0.73 on the stronger side and −1.24 ± 1.20 mm on the weaker side in the P group. The ES group exhibited significantly smaller reductions in quadriceps muscle thickness compared to the P group (*p* < 0.05). Similar findings were maintained at the 2-week follow-up (*p* < 0.05).

### Changes in quadriceps muscle strength

**Figure 3:**
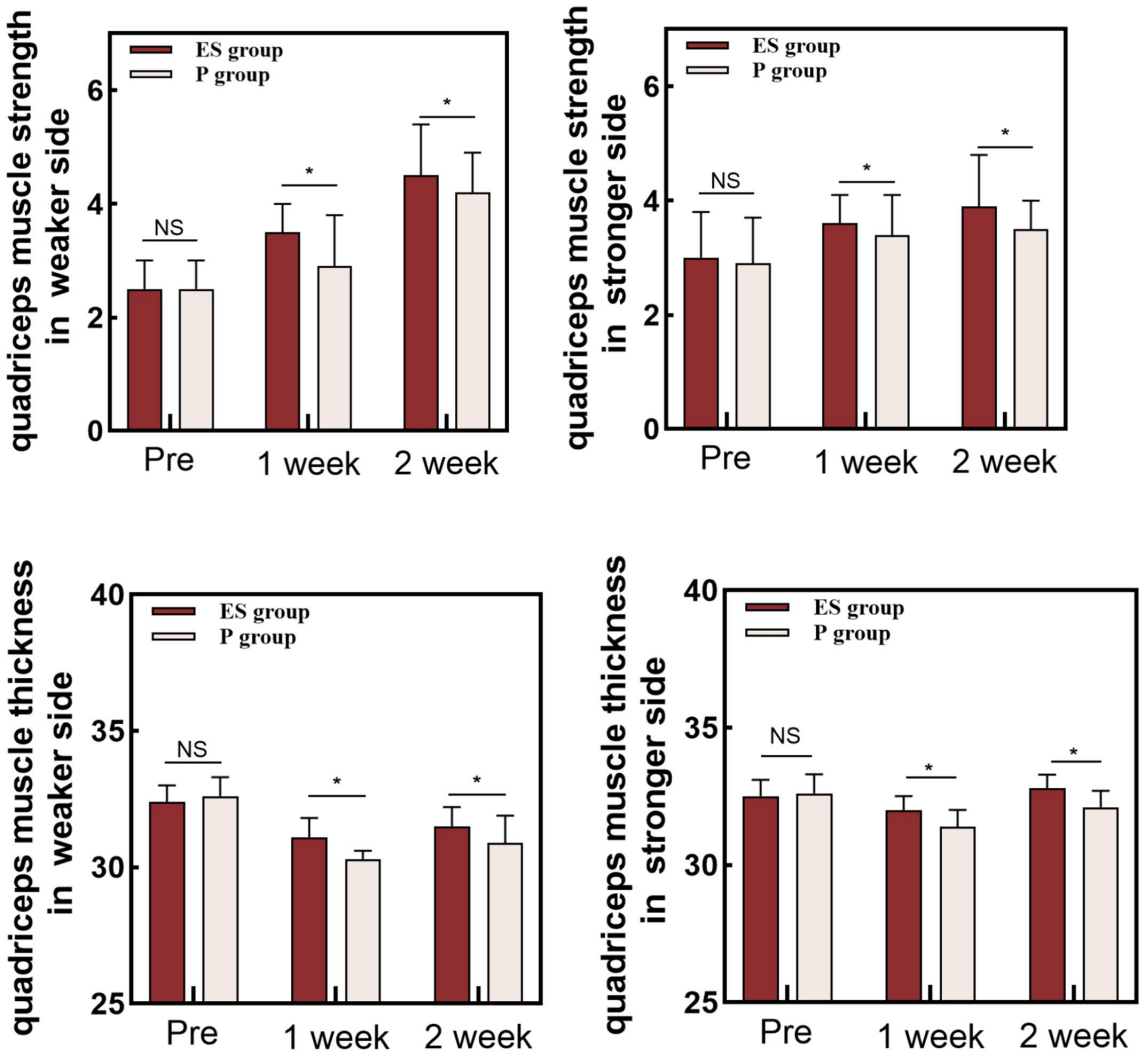
Outcomes of quadriceps muscle thickness and muscle strength at pretreatment, 1-week, and 2-week follow-up visits.

In the ES group, changes in quadriceps muscle strength on the stronger side were 0.93 ± 0.2 and 0.71 ± 0.4, and on the weaker side were 0.7 ± 0.39 and 0.4 ± 0.2 at the 1- and 2-week follow-up visits, respectively. In the P group, changes in quadriceps muscle strength on the stronger side were 0.38 ± 0.2 and 0.47 ± 0.3, while changes on the weaker side were 0.5 ± 1.1 and 0.2 ± 0.1 at the 1- and 2-week follow-up visits, respectively. The ES group demonstrated significantly greater improvement in quadriceps muscle strength on both sides at both follow-up time points compared to the P group (*p* < 0.05). To better illustrate the changes in quadriceps muscle thickness and muscle strength, these findings are presented in Figure 3.

## Discussion

Incomplete cervical SCI is a common condition that can lead to lower limb muscle atrophy, particularly in the quadriceps, due to disuse (16). In 2017, Grumbles et al. (17) analyzed 14 human SCI tissue samples from The Miami Project to Cure Paralysis tissue bank and reported that direct mechanical trauma may cause primary cellular injury, which may result in apoptotic, necrotic, and/or excitotoxic motoneuron death. Approximately 54% of motoneurons are lost after cervical SCI, leading to severe triceps brachii and quadriceps muscle atrophy in approximately 70% of patients. To date, numerous studies have reported that physiotherapy, including resistance training, is widely used to address muscle atrophy. Lu et al. (18) conducted a meta-analysis of 26 comparative studies and found that resistance training combined with other forms of exercise, such as balance, endurance, and aerobic training, significantly improved muscle strength and overall physical performance. Similarly, Sañudo et al. (19) also reported that 18 elderly adults who underwent flywheel resistance exercise demonstrated significantly greater improvements in knee flexion torque and walking speed compared to controls who received no intervention (*p* < 0.05).

However, patients with incomplete cervical SCI may find that their weaker limbs are unable to effectively engage in resistance training. As a result, several researchers have proposed ES as an effective intervention for muscle atrophy in patients with SCI (20). Oo et al. (21) assessed 16 patients with subacute SCI, with 8 receiving transcutaneous electrical nerve stimulation (TENS group) and 8 serving as controls. After 3 weeks of treatment, the TENS group demonstrated significantly greater improvements in composite spasticity scores, muscle tone scores, and ankle jerk scores compared to the control group (*p* < 0.05). Similarly, Giangregorio et al. (22) studied 34 patients with chronic incomplete SCI and found that those who received functional ES significantly showed significant improvements in lower-limb muscle cross-sectional area compared to controls (*p* < 0.05). Despite these findings, few studies have specifically examined the effects of ES on lower limb function in patients with subacute incomplete cervical SCI.

In this study, clinical outcomes were compared between patients with subacute incomplete cervical SCI who underwent ES and those who received traditional physiotherapy. Baseline characteristics, including age, height, weight, body mass index, gender, and ASIA scores, were similar between the groups (*p* > 0.05). Moreover, no significant pretreatment differences were observed in quadriceps muscle thickness or muscle strength. However, significantly greater improvements in quadriceps muscle thickness and muscle strength were observed in the ES group at both 1-week and final follow-up visits (*p* < 0.05). Further analysis revealed that although quadriceps muscle thickness decreased in both groups, the reduction was significantly less pronounced in the ES group. For the weaker side, changes in quadriceps muscle thickness were significantly smaller in the ES group than in the P group at both follow-up visits (−0.49 ± 0.81 mm vs. − 1.24 ± 1.2 mm at 1 week; −1.08 ± 0.68 mm vs. − 1.24 ± 0.82 mm at 2 weeks; *p* < 0.05). Despite decreased quadriceps muscle thickness in both groups, muscle strength significantly improved at both 1- and 2-week follow-up visits (*p* < 0.05). Several mechanisms may explain these findings. First, ES directly activates motor neurons, restores the recruitment of high-threshold motor units, and increases muscle contractions, which significantly improves muscle recovery (23, 24). Second, repeated muscle contractions promote metabolism, improve circulation, and reduce edema. This may contribute to the reduction in ultrasound-measured muscle thickness while enhancing functional muscle tissue quality (25). Similarly, Bochkezanian et al. (26) reported that ES strength training can promote metabolically active lean muscle mass and improve muscle force. Additionally, ES may help counteract protein breakdown associated with muscle denervation and disuse while increasing protein synthesis.

A key innovation of this study is its focus on patients with subacute incomplete cervical SCI. In contrast, most conventional ES therapies have primarily been used to treat patients with chronic lower limb muscle atrophy associated with lumbar intervertebral disk herniation (26). This study specifically evaluated the therapeutic effects of ES on acute disuse muscle atrophy during the subacute recovery phase. This approach may help patients recovering from cervical SCI surgery in regaining lower limb muscle function more rapidly and effectively. This could lead to earlier ambulation and improving overall walking recovery. The rationale for performing ES twice a day, every day, is based on the following: Traditionally, skeletal muscles require approximately 72 h of recovery after resistance training to avoid excessive fatigue and damage (27). However, there are differences between muscle contractions induced by ES and voluntary contractions: The motor unit pattern activated by ES is “fixed in space and synchronous in time.” However, ES training usually employs a relatively low intensity (mostly non-damaging and sub-maximal intensity aimed at preventing atrophy), and each stimulation session is relatively short. Therefore, a 48-h interval between sessions may not provide sufficient total training stimulation to counteract the rapid progression of denervation atrophy. For example, Zange et al. (28) reported administering ES for 20 min, twice a day, in the patient’s soleus muscle.

## Conclusion

This study investigated the effects of ES on quadriceps muscle atrophy and lower limb muscle strength in patients with subacute incomplete cervical SCI. The findings demonstrated that ES is more effective than traditional physiotherapy in improving quadriceps muscle preservation and enhancing lower limb muscle strength. These results support ES as a promising rehabilitation strategy for minimizing acute disuse muscle atrophy and promoting functional recovery in this patient population.

### Limitations

There are several limitations in this study. First, the sample size was small, and the follow-up time was short. Second, although it would have been beneficial to measure the cross-sectional perimeter and the degree of fat infiltration of the muscle using magnetic resonance imaging (MRI), this was a retrospective study and MRI scans were not performed previously. Third, the nutrient status and dietary intake were not recorded, as these factors may have an effect on muscle strength and thickness. Moreover, the bone densitometry (DEXA) was not conducted due to the retrospective nature of the study, which could have provided a more sensitive and accurate correlation between muscle characteristics and clinical therapy. Finally, multi-center, prospective large-sample, long-term follow-up randomized controlled trials need to be conducted to further confirm the effect of ES on subacute incomplete cervical SCI patients. Addtionally, a sham ES group should be considered in future studies to enhance the scientific validity of the results.

## Data Availability

The original contributions presented in the study are included in the article/Supplementary material, further inquiries can be directed to the corresponding author.
